# *MICB* Allele Genotyping on Microarrays by Improving the Specificity of Extension Primers

**DOI:** 10.1371/journal.pone.0142467

**Published:** 2015-11-16

**Authors:** In-Cheol Baek, Jung-Pil Jang, Eun-Jeong Choi, Tai-Gyu Kim

**Affiliations:** 1 Department of Microbiology, College of Medicine, The Catholic University of Korea, Seoul, Korea; 2 Hematopoietic Stem Cell Bank, College of Medicine, The Catholic University of Korea, Seoul, Korea; University of Malaya, MALAYSIA

## Abstract

Major histocompatibility complex (MHC) class I chain-related gene B (*MICB*) encodes a ligand for activating NKG2D that expressed in natural killer cells, γδ T cells, and αβ CD8^+^ T cells, which is associated with autoimmune diseases, cancer, and infectious diseases. Here, we have established a system for genotyping *MICB* alleles using allele-specific primer extension (ASPE) on microarrays. Thirty-six high quality, allele-specific extension primers were evaluated using strict and reliable cut-off values using mean fluorescence intensity (MFI), whereby an MFI >30,000 represented a positive signal and an MFI <10,000 represented a negative signal. Eight allele-specific extension primers were found to be false positives, five of which were improved by adjusting their length, and three of which were optimized by refractory modification. The *MICB* alleles (*002:01, *003, *005:02/*010, *005:03, *008, *009N, *018, and *024) present in the quality control panel could be exactly defined by 22 allele-specific extension primers. *MICB* genotypes that were identified by ASPE on microarrays were in full concordance with those identified by PCR-sequence-based typing. In conclusion, we have developed a method for genotyping *MICB* alleles using ASPE on microarrays; which can be applicable for large-scale single nucleotide polymorphism typing studies of population and disease associations.

## Introduction

The product of major histocompatibility complex (MHC) class I chain-related gene B (Gene ID: 4277; *MICB*) is expressed as three extracellular domains, a transmembrane segment, and a carboxy-terminal cytoplasmic tail [1]. The flexible extracellular domains—α1, α2, and α3—together serve as a ligand for activating NKG2D that is expressed on natural killer (NK) cells, γδ T cells, and αβ CD8^+^ T cells [2]. As shown in the IMGT/HLA database (http://www.ebi.ac.uk/imgt/hla), over 40 polymorphic alleles have been found in exon 2 to 6 of *MICB*. Specific polymorphisms are associated with autoimmune diseases, cancer, and infectious diseases, as well as with the success of hematopoietic stem cell transplantation. For example, MICB*004 allele is significantly associated with susceptibility to rheumatoid arthritis in Spanish Caucasians [3], MICB*005:02 allele is negatively associated with cervical cancer in the Thai population [4], and MICB*008 allele (designated as MICB*0106 allele in 2004) is positively associated with ulcerative colitis in the Han Chinese population of central China [5]. MICB*008 allele is also associated with a reduction in severity of dengue fever [6]. In addition, MICB*008 and *002 alleles are associated with susceptibility to celiac disease in Europeans [7]. Moreover, recipients in need of a transplant had significantly improved survival probabilities when *MICA* and *MICB* alleles matched between the donor and recipient [8].

In previous studies, genotyping *MICB* alleles has been carried out using polymerase chain reaction with sequence-specific primers (PCR-SSP) and PCR-sequence-based typing (PCR-SBT) [9, 10]. Methods for detecting single nucleotide polymorphisms (SNPs) on a microarray platform include single base extension (SBE), allele-specific primer extension (ASPE), oligonucleotide ligation, allele-specific oligonucleotide direct hybridization, allele-specific cleavage of a flap endonuclease, and 5′ nuclease assay [11–13]. In this study, we developed a high-throughput genotyping system for *MICB* alleles by optimizing allele-specific extension primers for ASPE on glass slides.

## Materials and Methods

### Genomic DNA extraction

Genomic DNA was extracted from peripheral blood samples taken from 200 healthy Korean individuals (staff and students of the College of Medicine, The Catholic University of Korea) using an AccuPrep Genomic DNA Extraction Kit (Bioneer Corporation; Daejeon, Korea), following the manufacturer’s instructions. Briefly, the cells were lysed in 100 μL K buffer (50 mmol/L KCl, 10 mmol/L Tris-HCl (pH 8.3), 2.5 mmol/L MgCl_2_, 0.5% Tween 20, and 100 μg/mL Proteinase K) for 60 min at 56°C and then inactivated for 10 min at 95°C. After extraction, the DNA concentration was adjusted to 100 ng/μL and the resulting DNA samples were used as the PCR template in the genotyping assays.

In the UCLA International KIR and MICA Exchange Report program, 95 DNA samples for KIR Exchange (KDNA # 0021, 0038 ~ 0067, 0069 ~ 0084, 0087 ~ 0109, 0111 ~ 0118, 0120 ~ 0136) and 68 DNA samples for MICA Exchange (MICA#025 ~ 092) were used for *MICB* genotyping by PCR-SBT. Six samples among them were defined to be MICB*005:01, *005:06 and *024 alleles, which did not find in Korean population and included to establish *MICB* genotyping on microarrays (Table 1).

**Table 1:** Panel for quality controls of ASPE on microarrays.

Sample no.^¶^	*MICB* alleles by PCR-SBT		Population
CHB-4	*004:01:01	*004:01:01	Korean
CHB-13	*002:01:01	*005:03	Korean
CHB-17	*002:01:01	*008	Korean
CHB-19	*002:01:01	*003	Korean
CHB-38	*002:01:01	*004:01:01	Korean
CHB-47	*003	*008	Korean
CHB-70	*005:02	*009N	Korean
CHB-72	*002:01:01	*014	Korean
CHB-73	*002:01:01	*018	Korean
CHB-77	*002:01:01	*019	Korean
CHB-78	*004:01:01	*014	Korean
CHB-84	*003	*005:03	Korean
KDNA # 0064	*005:03	*005:01	Asian Indian
KDNA # 0124	*005:02/*010	*005:06	Caucasian
KDNA # 0128	*005:02/*010	*005:06	Caucasian
MICA#036	*004:01	*024	Black
MICA#047	*005:02/*010	*005:01	Hispanic
MICA#88	*005:02/*010	*005:01	Black

^¶^CHB is abbreviation of the Catholic Hematopoietic Stem Cell Bank (http://www.chscb.com).

### Ethics statement

The study was approved by the Catholic University Institutional Review Board (IRB), with written informed consent from all subjects (Institutional Review Board number: MC13SISI0126).

### Preparation of microarrays

The matched control extension primers define as the N0 group were designed to match the *MICB* sequence without SNPs. Mismatched control extension primers were designed to contain artificial mismatches at the 1st, 2nd, 3rd, and 4th base from the 3′ ends of the N0 extension primers, and were called the N1, N2, N3, and N4 groups, respectively. Each group consisted of four primers containing an adenine, cytosine, guanine, or thymine (A, C, G, or T) as the mismatch at the 3′ end (Table 2).

**Table 2:** Control extension primer set for optimal conditions of ASPE on microarrays.

Groups according to mismatches from 3′ends	Names of control extension primers	Sequence (5′-3′)	Region^¶^	Length (bp)	Tm (°C)^†^	GC contents (%)
N0 (matched)						
	A0	GAGAATGGGCAAGACCTCA	7623–7641	19	51.1	52.6
	C0	TGTGCAGTCAGGGTTTCTC	7472–7490	19	51.1	52.6
	G0	GCAGTGGGGGGATGTCCTG	8967–8985	19	57.6	68.4
	T0	TGGATGGTCAGCCCTTCCT	7504–7522	19	53.2	57.9
N1 (mismatched 1st base from 3′ends)						
	A1	GAGAATGGGCAAGACCTC**G**	7623–7641	19	53.2	57.9
	C1	TGTGCAGTCAGGGTTTCT**A**	7472–7490	19	48.9	47.4
	G1	GCAGTGGGGGGATGTCCT**A**	8967–8985	19	55.4	63.2
	T1	TGGATGGTCAGCCCTTCC**G**	7504–7522	19	55.4	63.2
N2 (mismatched 2nd base from 3′ends)						
	A2	GAGAATGGGCAAGACCT**T**A	7623–7641	19	48.9	47.4
	C2	TGTGCAGTCAGGGTTTC**G**C	7472–7490	19	53.2	57.9
	G2	GCAGTGGGGGGATGTCC**G**G	8967–8985	19	59.7	73.7
	T2	TGGATGGTCAGCCCTTC**A**T	7504–7522	19	51.1	52.6
N3 (mismatched 3rd base from 3′ends)						
	A3	GAGAATGGGCAAGACC**G**CA	7623–7641	19	53.2	57.9
	C3	TGTGCAGTCAGGGTTT**A**TC	7472–7490	19	48.9	47.4
	G3	GCAGTGGGGGGATGTC**A**TG	8967–8985	19	55.4	63.2
	T3	TGGATGGTCAGCCCTT**A**CT	7504–7522	19	51.1	52.6
N4 (mismatched 4th base from 3′ends)						
	A4	GAGAATGGGCAAGAC**A**TCA	7623–7641	19	48.9	47.4
	C4	TGTGCAGTCAGGGTT**G**CTC	7472–7490	19	53.2	57.9
	G4	GCAGTGGGGGGATGT**A**CTG	8967–8985	19	55.4	63.2
	T4	TGGATGGTCAGCCCT**G**CCT	7504–7522	19	55.4	63.2

^¶^References from IMGT/HLA databases [14].

^†^Calculation of melting temperature (Tm) is described in Materials and Methods

Underlines, a mismatched base, A, C, G, or T, utilizing binding errors of both purine and pyrimidine.

Microarrays were prepared as previously described [15]. Each of the *MICB* allele-specific extension primers (50 μmol/L), which were designed based on the sequences of the *MICB* polymorphisms with an additional 18 bp oligo(dT) spacer at the 5′ end, was dissolved in a buffer containing 350 mmol/L sodium bicarbonate (pH 9.0) (Tables 3 and 4). The primers were then spotted onto standard microscope glass slides coated with aldehyde (SCHOTT; Jena, Germany) using a MicroGridII (BioRobotics; Cambridge, MA, USA) controlled with an MCM-310 operating system. The primers were immobilized as NH_2_-modified oligonucleotides. Distilled water was used to hydrate the primers on the slides at 25°C for 1 h in a humid chamber, and then the slides were baked at 120°C for 1 h.

**Table 3:** First designed 36 allele-specific extension primers.

Names of allele-specific extension primers	Sequence (5′-3′)	SNPs found in Korean*	Region^¶^	Length	Tm (°C)^†^	GC contents (%)	Quality of primer^§^	*MICB* alleles				
								Frequency >1%	Frequency <1%	Alleles found in the other populations	Alleles determined by two allele-specific primers	
*MICB* 86G	CAGAGCCCCACAGTCTTCG	G	7420–7438	19	55.4	63.2	ND					
*MICB* 86A	CAGAGCCCCACAGTCTTCA	−	7420–7438	19	53.2	57.9	ND		*023			
*MICB* 116G	TGGTGCTGTCCCAGGATGG	G	7450–7468	19	55.4	63.2	ND					
*MICB* 116A	TGGTGCTGTCCCAGGATGA	−	7450–7468	19	53.2	57.9	ND		*001			
*MICB* 163C	GGACATCTGGATGGTCAGC	C	7497–7515	19	53.2	57.9	+					
*MICB* 163T	GGACATCTGGATGGTCAGT	−	7497–7515	19	51.1	52.6	−			*024		
*MICB* 203C	AGAAACGCAGGGCAAAGCC	C	7537–7555	19	53.2	57.9	ND					
*MICB* 203A	AGAAACGCAGGGCAAAGCA	−	7537–7555	19	51.1	52.6	ND		*011			
*MICB* 223G	AGGGACAGTGGGCAGAAG	G	7558–7575	18	52.6	61.1	+					
*MICB* 223A	CAGGGACAGTGGGCAGAAA	A	7557–7575	19	53.2	57.9	+	*004:01				
*MICB* 238A	GAARATGTCCTGGGAGCTA	A	7572–7590	19	51.1	47.4	−				*018	
*MICB* 238G	GAARATGTCCTGGGAGCTG	G	7572–7590	19	53.2	52.6	−	*002:01				*019
*MICB* 263A	GGACACAGAGACCGAGGA	A	7598–7615	18	52.6	61.1	ND					
*MICB* 263G	GGACACAGAGACCGAGGG	−	7598–7615	18	54.9	66.7	ND		*022			
*MICB* 314A	GACCCTGACTCATATCAAGGA	A	7646–7666	21	52.4	47.6	ND					
*MICB* 314G	ACCCTGACTCATATCAAGGG	G	7647–7666	20	51.8	50.0	ND		*012			
*MICB* 363C	GATTAGGGTCTGTGAGATC	C	7968–7986	19	48.9	47.4	+					
*MICB* 363G	GATTAGGGTCTGTGAGATG	−	7968–7986	19	48.9	47.4	−	*008				
*MICB* 406G	TCCCGGCATTTCTACTACG	G	8011–8029	19	51.1	52.6	+					*019
*MICB* 406A	TCCCGGCATTTCTACTACA	A	8011–8029	19	48.9	47.4	−	*002:01			*018	
*MICB* 577C	CTGCCTGCAGAAACTACAGC	C	8181–8201	20	53.8	55.0	+					
*MICB* 577T	TGCCTGCAGAAACTACAGT	T	8182–8201	19	48.9	47.4	−	*009N				
*MICB* 635C	CCCCCATGGTGAATGTCAC	C	8831–8849	19	53.2	57.9	+					
*MICB* 635T	CCCCCATGGTGAATGTCAT	T	8831–8849	19	51.1	52.6	−	*003				
*MICB* 643G	GGTGAATGTCAYCTGCAGCG	G	8838–8857	20	55.9	55.0	ND					
*MICB* 643A	GGTGAATGTCACCTGCAGCA	−	8838–8857	20	53.8	55.0	ND		*006/*015			
*MICB* 699G	TTCCAGCTTCTATCCCCGG	G	8895–8913	19	53.2	57.9	+					
*MICB* 699A	TTCCAGCTTCTATCCCCGA	A	8895–8913	19	51.1	52.6	−	*005:03				
*MICB* 762G	CAACACCCAGCAGTGGGGG	G	8958–8976	19	57.6	68.4	+					
*MICB* 762T	CAACACCCAGCAGTGGGGT	−	8958–8976	19	55.4	63.2	+			*005:06		
*MICB* 836G	GCCAAGGAGAGGAGCAGAG	G	9032–9050	19	55.4	63.2	ND					
*MICB* 836A	GCCAAGGAGAGGAGCAGAA	−	9032–9050	19	53.2	57.9	ND		*007			
*MICB* 870C	GGAACACAGCGGGAATCAC	C	9066–9084	19	53.2	57.9	+					
*MICB* 870T	GGAACACAGCGGGAATCAT	−	9066–9084	19	51.1	52.6	+			*005:01		
*MICB* 871G	GAACACAGCGGGAATCACG	G	9067–9085	19	53.2	57.9	+					
*MICB* 871A	GAACACAGCGGGAATCACA	A	9067–9085	19	51.1	52.6	+	*014				

*-, not present in Korean

^¶^References from IMGT/HLA databases [14].

^§^+, successful primer

-, failed primer; ND, not determined in our control panel (Table 1).

Underlines, *MICB* polymorphic sites.

**Table 4:** Allele-specific extension primers improved by length and refractory modification.

	Names of allele-specific extension primers	Sequence (5′-3′)	Region^¶^	Length	Adjustment of bases	Tm (°C)^†^	GC contents (%)	Quality of primer
**Length modification**								
	*MICB* 163T	GGACATCTGGATGGTCAGT	7497–7515	19	0	51.1	52.6	−
	*MICB* 163T-1	ACATCTGGATGGTCAGT	7449–7515	17	−2	44.6	47.1	+
	*MICB* 238G	GAARATGTCCTGGGAGCTG	7572–7590	19	0	53.2	52.6	−
	*MICB* 238G-1	ARATGTCCTGGGAGCTG	7574–7590	17	−2	49.5	52.9	−
	*MICB* 238G-2	ATGTCCTGGGAGCTG	7576–7590	15	−4	44.7	60.0	+
	*MICB* 577T	TGCCTGCAGAAACTACAGT	8181–8201	19	0	48.9	47.4	−
	*MICB* 577T-1	CCTGCAGAAACTACAGT	8183–8201	17	−2	44.6	47.1	−
	*MICB* 577T-2	TGCAGAAACTACAGT	8185–8201	15	−4	36.5	40.0	+
	*MICB* 635T	CCCCCATGGTGAATGTCAT	8831–8849	19	0	51.1	52.6	−
	*MICB* 635T-1	CCCATGGTGAATGTCAT	8833–8849	17	−2	44.6	47.1	+
	*MICB* 699A	TTCCAGCTTCTATCCCCGA	8895–8913	19	0	51.1	52.6	−
	*MICB* 699A-1	CCAGCTTCTATCCCCGA	8897–8913	17	−2	49.5	58.8	−
	*MICB* 699A-2	AGCTTCTATCCCCGA	8899–8913	15	−4	41.9	53.3	+
**Refractory modification**								
	*MICB* 238A	GAARATGTCCTGGGAGCTA	7572–7590	19	0	51.1	47.4	−
	*MICB* 238A-1	CAGAARATGTCCTGGGAGCTA	7570–7590	21	+2	54.4	47.6	−
	*MICB* 238A-2	GGCAGAARATGTCCTGGGAGCTA	7568–7590	23	+4	58.8	52.2	−
	*MICB* 238A-3	GGCAGAARATGTCCTGGGAGCTAA	7568–7591	24	+5	59.1	50.0	+
	*MICB* 363G	GATTAGGGTCTGTGAGATG	7968–7986	19	0	48.9	47.4	−
	*MICB* 363G-1	GAGATTAGGGTCTGTGAGATG	7966–7986	21	+2	52.4	47.6	−
	*MICB* 363G-2	GAGATTAGGGTCTGTGAGATGC	7966–7987	22	+3	54.8	50.0	+
	*MICB* 406A	TCCCGGCATTTCTACTACA	8011–8029	19	0	48.9	47.4	−
	*MICB* 406A-1	GCTCCCGGCATTTCTACTACA	8009–8029	21	+2	54.4	52.4	−
	*MICB* 406A-2	GCTCCCGGCATTTCTACTACAA	8009–8030	22	+3	54.8	50.0	−
	*MICB* 406A-3	GCTCCCGGCATTTCTACTACAAT	8009–8031	23	+4	55.3	47.8	+

^¶^References from IMGT/HLA databases [14].

^§^+, successful primer

-, failed primer.

Underlines, *MICB* polymorphic sites.

Next, to deoxidize the aldehyde residue, the slides were washed for 30 min, first with 0.2% sodium dodecyl sulfate (Sigma; Dorset, UK), followed by distilled water, and then with an NaBH_4_ solution containing 1 g NaBH_4_, 300 mL PBS, and 100 mL EtOH. Finally, the slides were rinsed with 0.2% sodium dodecyl sulfate followed by distilled water and then dried. The processed slides were stored at −70°C in a silica-gel box.

### Amplification of *MICB*

Primer sets were designed to amplify *MICB* polymorphisms, which are present in exon 2, 3, 4, and 5 of the *MICB* gene. The forward primers were modified to have a phosphate group (P) at the 5′-end. The following primers were used in each single tube: sense 1, 5′-P-CAATGTGAAGTTATTTCCAGGAAGAAG-3′ from positions 7369 to 7395); antisense 1, 5′-CCAGGGTCGGTACCTGTTCT-3′ (from positions 8230 to 8249) for *MICB* exon 2 and 3; sense 2, 5′-P-CTGTTCCCTGCATCTCCCTTAGA-3′ (from positions 8765 to 8787); antisense 2, 5′-CCCATCTCCAGAAACTGTCCCC-3′ (from positions 9346 to 9368) for *MICB* exon 4 and 5 [14]. PCR was carried out in a reaction volume of 40μL containing 500 ng genomic DNA, 1× buffer (60 mmol/L Tris-Cl, 15 mmol/L ammonium sulfate, 100 mmol/L MgCl_2_), 200 μmol/L dATP/dGTP/dCTP, 200 μmol/L dTTP/dUTP (dT:dU = 7:3), 4U Taq DNA polymerase (Bioprince; Enzynomics; Daejeon, Korea) and 10 μmol/L of each primer. The reactions were amplified using a Mycycler Thermo Cycler (Bio-rad Inc.; CA, USA) with the following conditions: initial denaturation of 98°C for 20 s; 8 cycles of denaturation at 98°C for 5 s, annealing at 65°C for 30 s, and extension at 72°C 30 s; followed by 37 cycles of denaturation at 98°C for 5 s, annealing at 60°C for 30 s, and extension at 72°C for 30 s; and followed by a final extension at 72°C for 10 min. Then, the reactions were held at 20°C.

### Primer extension on glass slides

The PCR products were treated with 10× λ exonuclease buffer containing 3.5U λ exonuclease (Fermentas; Burlington, Canada) and 1.75U shrimp alkaline phosphate (Qiagen; Hamburg, Germany). After the PCR products had been cleaved into approximately 50-bp fragments, they were hybridized with a mixture that contained 25× Themo Sequenase buffer (Enzymonics; Daejeon, Korea), 3.5U Thermo Sequenase (diluted 1:8 in Sequenase dilution buffer; Enzynomics; Daejeon, Korea), and 0.1 mmol/L each of both dATP/dTTP/dGTP and Cy5-dCTP at 60°C for 1 h. To remove non-specific primers, the slides were washed for five minutes with a solution containing 0.1% Alconox and then rinsed twice with distilled water, for 5 min each time.

**Fig 1:**
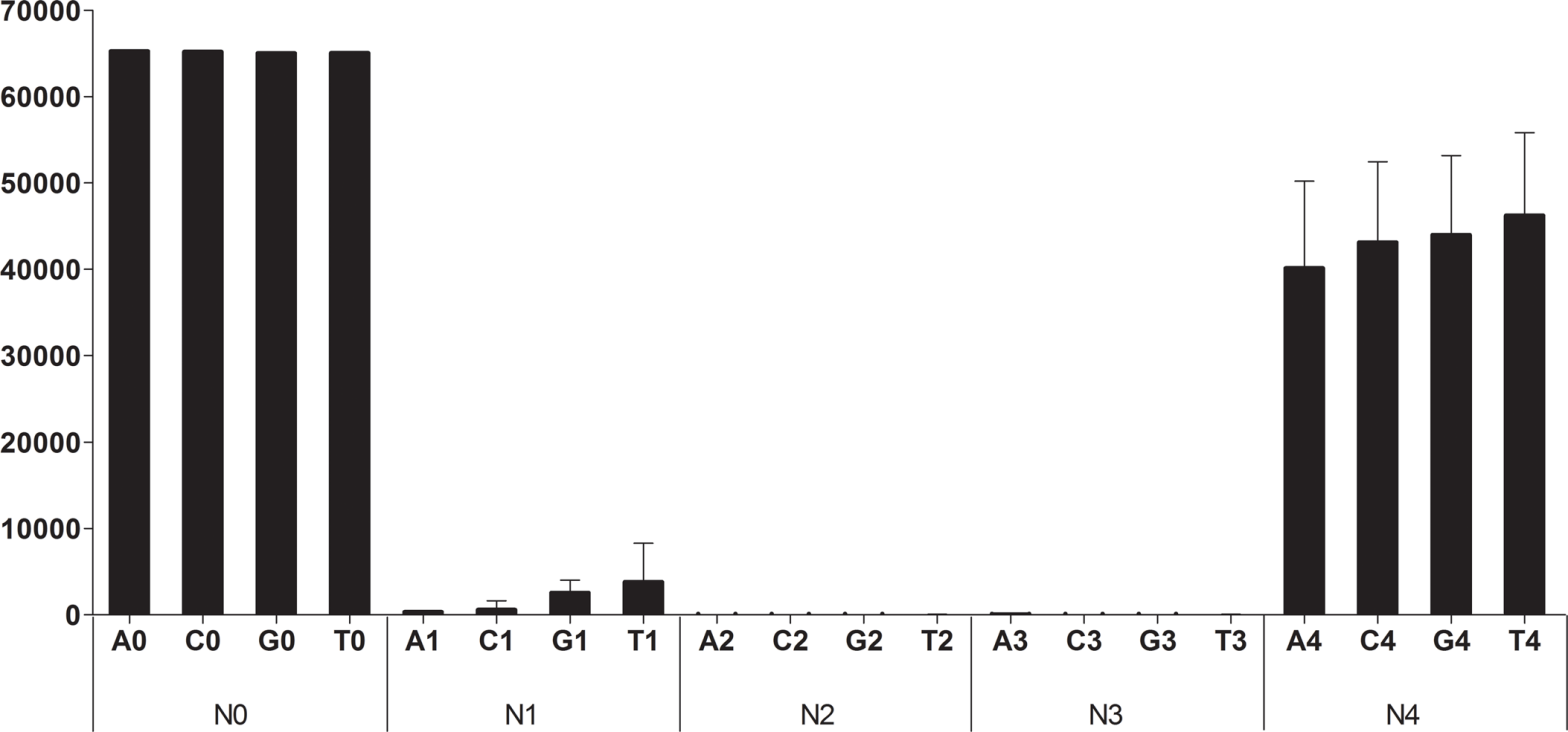
The optimization of ASPE on microarrays for quality controls of allele-specific extension primers using control extension primers. The N0 group of matched control extension primers was made to match the sequences of regions without SNPs in the exon of the *MICB* gene. Mismatched control extension primers were made to contain artificial mismatches at the 1st, 2nd, 3rd, and 4th bases from the 3′ ends of the N0 extension primers, and were defined as the N1, N2, N3 and N4 groups. Each control primer group consisted of four primers that contained adenine, cytosine, guanine, or thymine (A, C, G, or T) at the 3′ end. MFI, mean fluorescence intensity.

The data were obtained using the EasyScan software after scanning with NanoDscan (Nanostorage; Seoul, Korea) and then analyzed for genotyping using Microsoft Excel. The background mean fluorescence intensity (MFI) was subtracted from the MFI of each primer; cut-off values were determined using quality control samples (Fig 1).

### PCR-SBT for *MICB*

*MICB* primers were designed as previously described [16]. PCR was carried out in a reaction volume of 10 μL containing 200 ng genomic DNA, 1× buffer (60 mmol/L Tris-Cl, 15 mmol/L Ammonium Sulfate, 100 mmol/L MgCl_2_), 200 μmol/L dATP/dGTP/dCTP/dTTP, 0.6 U Taq DNA polymerase (Enzynomics; Daejeon, Korea), bovine serum albumin, Cresol red (in 28% sucrose) and 3.7 μmol/L of each primer. The cycling conditions were as follows: 95°C for 5 min; then 19 cycles of 94°C for 45 s, 65°C for 45 s (a reduction of −0.5°C per cycle), and 72°C for 2 min; then 13 cycles of 94°C for 45 s, 55°C for 45 s, and 72°C for 2 min; and finally, 72°C for 10 min. Then, the reactions were held at 20°C.

The *MICB* exon 6 primers were designed to differentiate MICB*005:02 allele from MICB*010 allele in exon 6, with the forward primer as 5′-GGGCAACTGAAGAGAGAAAAG-3′ and the reverse primer as 5′-CAGGAGCAGTCGTGAGTTTG-3′. The PCR conditions were as follows: an initial denaturation of 95°C for 5 min; 30 cycles of denaturation at 95°C for 30 s, annealing at 60°C for 30 s, and extension at 72°C for 30 s; and a final extension at 72°C for 10 min. Then the reactions were held at 20°C.

Purified PCR products were sequenced using an ABI 377 DNA sequencer; the *MICB* primers for PCR amplification were also used as the sequencing primers. The sequencing data were analyzed using the ABI Factura software and the ABI Sequence Navigator program (PE Biosystems; Mississauga, Canada).

### Calculation of melting temperature and statistical analysis

The melting temperature (Tm) for sequences longer than 13 bases was calculated as follows:

$$Tm=64.9+\frac{41\times (yG+zC-16.4)}{wA+xT+yG+zC}$$

W, x, y, and z indicate the number of adenine, thymine, guanine and cytosine (A, T, G, and C nucleotides), respectively, in the sequence.

The Tm and GC content in primers from the successful group of allele-specific extension primers were compared to those from the failed group using the unpaired Student’s *t*-test. We corrected for statistical analyses calculated from five or less observed scores using Fisher’s exact test.

## Results

### Control extension primers for quality control

For quality control, 20 extension primers were designed against sequences in *MICB* exon 2, 3, 4, and 5. The primers were 19 bp long, with a Tm of 53.0 ± 3.0°C, and a GC content of 57.4 ± 7.4% (Table 2). The MFI value of the N0 primers was higher than the maximum value (65,535 MFI) of N0 primers measured by the EasyScan-1 scanning machine, and the MFI value for the N4 primers was >30,000. By contrast, the N1 primer group produced a very weak signal, whereas N2 and N3 displayed refractory extension (Fig 1). As the N0 and N4 primers showed strong positive signals, they were used as positive controls, and the primers in the other groups were used as negative controls. Based on these results, we set very strict and reliable cut-off values for MFI: an MFI >30,000 was considered as a positive signal, and an MFI <10,000 was considered a negative signal. Using these cut-offs, it was possible to select high quality allele-specific extension primers.

### Optimization of allele-specific extension primers

The ability to discriminate *MICA* alleles from *MICB* alleles was verified using primer location and a cross over test. Primers for amplification of exons 2–5 of *MICB* alleles did not exist in the *MICA* gene (Gene ID: 100507436). The PCR products of *MICB* exons 2–5 did not showed any positive signals on the *MICA* allele-specific extension primers of microarray for *MICA* allele genotyping [17].

Eighteen samples including 13 *MICB* alleles previously identified in Koreans and other populations were selected as the quality control panel for ASPE on microarrays. We defined *MICB* alleles of 163 samples in the UCLA International KIR and MICA Exchange Report program. Among them, six samples including three *MICB* alleles of MICB*005:01, *005:06 and *024 alleles which did not find in Korean population were added in Table 1. *MICB* alleles of the other 157 samples were also found in Korean population. A validation of the amplification reaction has been performed for the alleles (MICB*002:01, *003, *004:01, *005:01, *005:02/*010, *005:03, *005:06, *008, *009N, *014, *018, *019, and *024 alleles) included in the quality control panel. To identify the 41 known *MICB* alleles, 36 allele-specific extension primers were designed with a length of 18 to 21 bases. Among these primers, the specificity and sensitivity of only 22 primers could be determined by the quality control panel used in this study (Table 3). Of these primers, 14 showed excellent sensitivity and specificity with a Tm of 52.9 ± 2.1°C and a GC content of 57.2 ± 5.1%. The eight failed primers had a Tm of 50.6 ± 1.5°C and a GC content of 50.0 ± 2.8%. The Tm and GC content were significantly different between the successful extension primer group and the failed primer group (*P* < 0.02 for each category).

**Fig 2:**
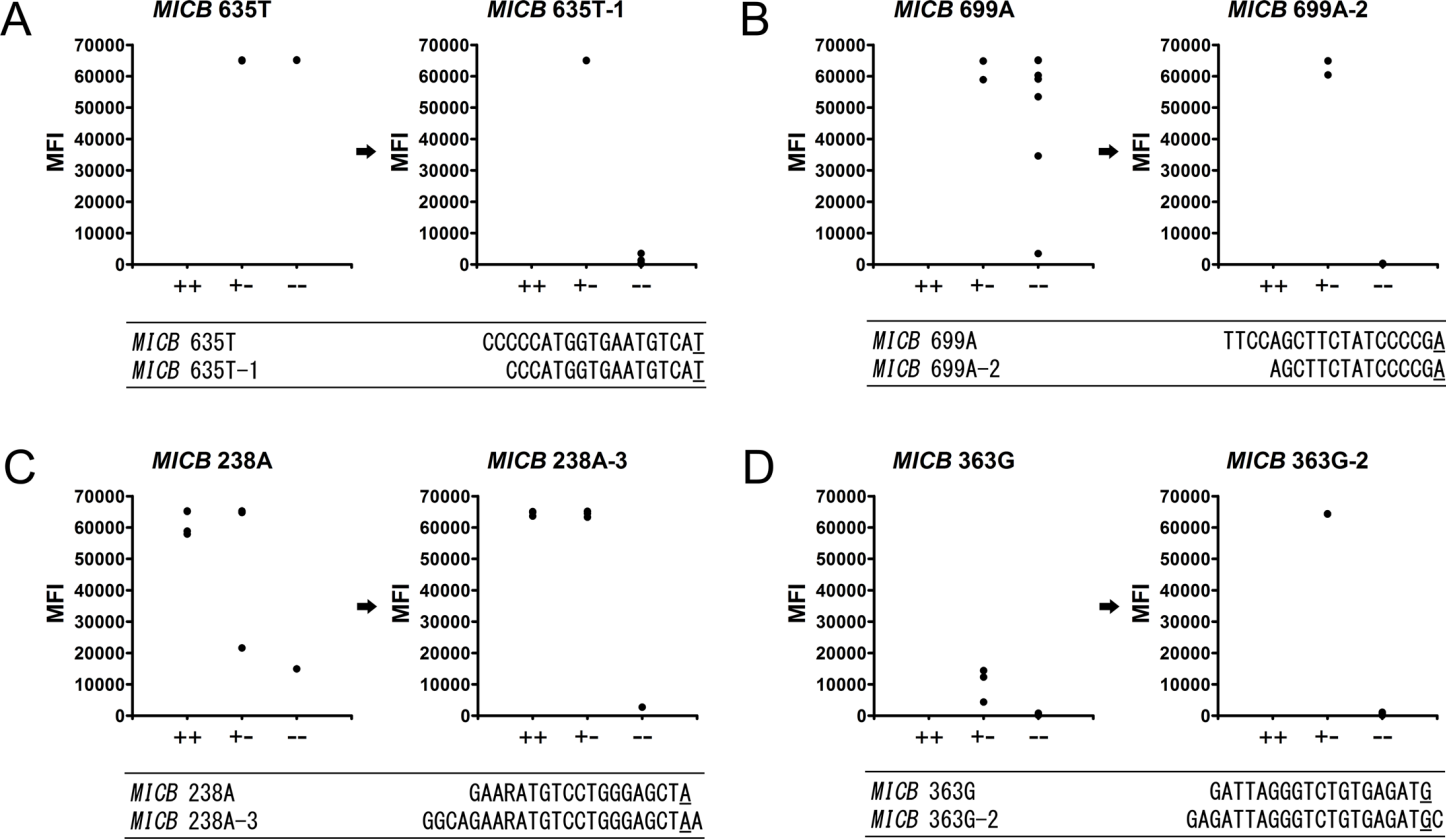
The optimal length and refractory modification for high quality allele-specific extension primers. We improved the quality of the primers by deleting two bases at the 5′ end of the previous primer (A), deleting four bases at the 5′ end (B), deleting four bases at the 5′ end and adding one base at the SNP (C), and adding two bases at the 5′ end and one base at the SNP (D). ‘+ +’, positive homozygote samples; ‘+ −’, heterozygote; ‘− −’, negative homozygote.

Of the eight failed primers, five were optimized by shortening the primer at the 5′ end to eliminate false positive signals, and the other three were optimized by correcting mismatches that were refractory to extension (Fig 2; Table 4). All the MICB*002:01, *003, *005:02/*010, *005:03, *008, *009N, *018, and *024 alleles present in our panel showed perfect matches by 22 allele-specific extension primers validated with the quality control panel after optimal modification of primers (Tables 3 and 4). Although alleles showing mismatches did not exist in our panel, rare alleles still need to be further defined. The MICB*005:02/*010 allele was verified by negative signals of 14 allele-specific primers matching the other 12 *MICB* alleles above. We speculate three were allele and genotype ambiguities not resolvable by ASPE in the above condition such as MICB*004:01/*020/*025/*026/*028, *005:06/*005:07/*005:08/*010/*021N/*027/*030 and *002:01/*029.

### Genotyping of *MICB* alleles

**Fig 3:**
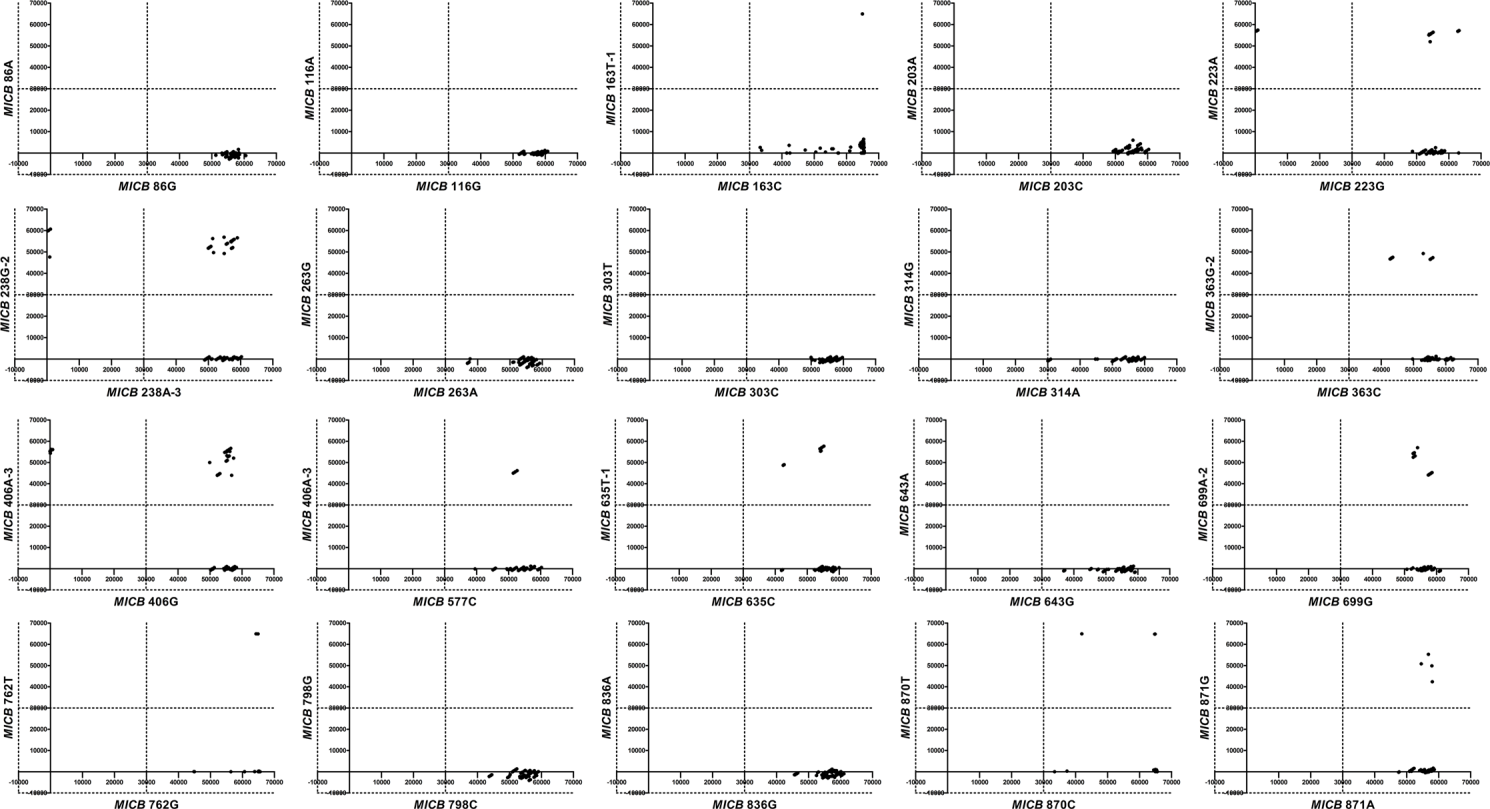
Scatter plots showing the genotype assignment of 18 SNPs for 200 healthy Koreans and 6 individuals of other populations. The allele-specific extension primers of each scatter plot are shown in Tables 3 and 4. *MICB* 223G, *MICB* 223A, *MICB* 238A-3, *MICB* 238G-2, *MICB* 406G, and *MICB* 406A-3 showed all three genotypes. *MICB* 163C, *MICB* 163T-1, *MICB* 363C, *MICB* 363G-2, *MICB* 577C, *MICB* 577T-2, *MICB* 635C, *MICB* 635T-1, *MICB* 762G, *MICB* 762T, *MICB* 870C, *MICB* 870T, *MICB* 871A and *MICB* 871G were one heterozygote and one homozygote. The others were one homozygote.

The ASPE on microarrays were evaluated using the arrays that genotyped the 18 SNPs in the *MICB* alleles. The samples of known *MICB* alleles from about 200 Koreans and six individuals of other populations were analyzed for genotyping. Scatter plots were resulted in numerical presentation about one clustering of three genotypes. The similar values between the signals from the normal allele-specific reactions were divided into three districts depending on the genotype; 20 SNPs were over 30,000 MFI (Fig 3). Scatter plots showed that the allele-specific reactions could be divided into three groups (223AA/AG/GG, 238AA/AG/GG, and 406AA/AG/GG). Another 8 SNP regions had one heterozygote and one homozygote (163CT/CC, 363CG/GG, 577CT/TT, 635CT/TT, 699AG/GG, 762GT/GG, 870CT/CC, and 871AG/GG). The other seven SNPs existed in the homozygous state (86GG, 116GG, 203CC, 263AA, 314AA, 643GG, and 836GG).

Analysis of *MICB* alleles using the 22 allele-specific extension primers resulted in 27 different combinations of SNPs (Table 5). The most common genotype was homozygous for MICB*005:02/*010 (n = 56), followed by MICB*005:02/*010/*004:01 (n = 32), *005:02/*010/*002:01 (n = 30), *005:02/*010/*005:03 (n = 16), *005:02/*010/*008 (n = 15), *005:02/*010/*009N (n = 8), *005:02/*010/*003 (n = 8), *002:01/*014 (n = 6), *002:01/*008 (n = 4), *004:01/*004:01 (n = 4), *002:01/*004:01 (n = 4), *002:01/*005:03 (n = 2), *003/*008 (n = 2), *005:03/*009N (n = 2), *002:01/*003 (n = 2), *005:02/*010/*005:01 (n = 2), *005:02/*010/*005:06 (n = 2) and*002:01/*002:01 (n = 2). The other 9 combinations were seen in only one sample each (*002:01/*018, *002:01/*019, *003/*005:03, *004:01/*014, *005:02/*010/*014, *005:03/*005:01, *003/*004:01, *003/*005:01 and *004:01/*024). Because this *MICB* alleles’ genotyping on microarrays with ASPE can discriminate the 18 SNPs among 28 SNPs known in *MICB* gene (Table 5), the other 10 SNPs of *MICB* did not detect and not discriminate all alleles, existed many ambiguous allele combinations. These SNPs are present only in very rare alleles which have been not reported in published papers and are not found in 163 samples of the UCLA International Exchange Report program. Therefore, it may have some limitations for application compared with direct sequencing method, but could be helpful for disease association studies as high-throughput and intermediate resolution technique.

**Table 5:** *MICB* alleles defined by ASPE on microarrays.

ASPE on microarrays			Names of allele-specific extension primers																	
			86	116	163	203	223	238	263	314	363	406	577	635	643	699	762	836	870	871
Allele 1	Allele 2	n = 206	A/G	A/G	C/T	A/C	A/G	A/G	A/G	A/G	C/G	A/G	C/T	C/T	A/G	A/G	G/T	A/G	C/T	A/G
*005:02/*010	*005:02/*010	56	GG	GG	**CC**	CC	**GG**	**AA**	AA	AA	**CC**	**GG**	**CC**	**CC**	GG	**GG**	**GG**	GG	**CC**	**GG**
*005:02/*010	*004:01	32	GG	GG	**CC**	CC	**AG**	**AA**	AA	AA	**CC**	**GG**	**CC**	**CC**	GG	**GG**	**GG**	GG	**CC**	**GG**
*005:02/*010	*002:01	30	GG	GG	**CC**	CC	**GG**	**AG**	AA	AA	**CC**	**AG**	**CC**	**CC**	GG	**GG**	**GG**	GG	**CC**	**GG**
*005:02/*010	*005:03	16	GG	GG	**CC**	CC	**GG**	**AA**	AA	AA	**CC**	**GG**	**CC**	**CC**	GG	**AG**	**GG**	GG	**CC**	**GG**
*005:02/*010	*008	15	GG	GG	**CC**	CC	**GG**	**AG**	AA	AA	**CG**	**AG**	**CC**	**CC**	GG	**GG**	**GG**	GG	**CC**	**GG**
*005:02/*010	*009N	8	GG	GG	**CC**	CC	**GG**	**AA**	AA	AA	**CC**	**GG**	**CT**	**CT**	GG	**GG**	**GG**	GG	**CC**	**GG**
*005:02/*010	*003	8	GG	GG	**CC**	CC	**GG**	**AA**	AA	AA	**CC**	**GG**	**CC**	**CT**	GG	**GG**	**GG**	GG	**CC**	**GG**
*002:01	*014	6	GG	GG	**CC**	CC	**GG**	**GG**	AA	AA	**CC**	**AA**	**CC**	**CC**	GG	**GG**	**GG**	GG	**CC**	**AG**
*002:01	*008	4	GG	GG	**CC**	CC	**GG**	**GG**	AA	AA	**CG**	**AA**	**CC**	**CC**	GG	**GG**	**GG**	GG	**CC**	**GG**
*004:01	*004:01	4	GG	GG	**CC**	CC	**AA**	**AA**	AA	AA	**CC**	**GG**	**CC**	**CC**	GG	**GG**	**GG**	GG	**CC**	**GG**
*002:01	*004:01	4	GG	GG	**CC**	CC	**AG**	**AG**	AA	AA	**CC**	**AG**	**CC**	**CC**	GG	**GG**	**GG**	GG	**CC**	**GG**
*002:01	*005:03	2	GG	GG	**CC**	CC	**GG**	**AG**	AA	AA	**CC**	**AG**	**CC**	**CC**	GG	**AG**	**GG**	GG	**CC**	**GG**
*003	*008	2	GG	GG	**CC**	CC	**GG**	**AG**	AA	AA	**CG**	**AG**	**CC**	**CT**	GG	**GG**	**GG**	GG	**CC**	**GG**
*005:03	*009N	2	GG	GG	**CC**	CC	**GG**	**AA**	AA	AA	**CC**	**GG**	**CT**	**CT**	GG	**AG**	**GG**	GG	**CC**	**GG**
*002:01	*003	2	GG	GG	**CC**	CC	**GG**	**AG**	AA	AA	**CC**	**AG**	**CC**	**CT**	GG	**GG**	**GG**	GG	**CC**	**GG**
*005:02/*010	*005:01	2	GG	GG	**CC**	CC	**GG**	**AA**	AA	AA	**CC**	**GG**	**CC**	**CC**	GG	**GG**	**GG**	GG	**CT**	**GG**
*005:02/*010	*005:06	2	GG	GG	**CC**	CC	**GG**	**AA**	AA	AA	**CC**	**GG**	**CC**	**CC**	GG	**GG**	**GT**	GG	**CT**	**GG**
*002:01	*002:01	2	GG	GG	**CC**	CC	**GG**	**GG**	AA	AA	**CC**	**AA**	**CC**	**CC**	GG	**GG**	**CC**	GG	**CC**	**GG**
*002:01	*018	1	GG	GG	**CC**	CC	**GG**	**AG**	AA	AA	**CC**	**AA**	**CC**	**CC**	GG	**GG**	**GG**	GG	**CC**	**GG**
*002:01	*019	1	GG	GG	**CC**	CC	**GG**	**GG**	AA	AA	**CC**	**AG**	**CC**	**CC**	GG	**GG**	**GG**	GG	**CC**	**GG**
*003	*005:03	1	GG	GG	**CC**	CC	**GG**	**AA**	AA	AA	**CC**	**GG**	**CC**	**CT**	GG	**AG**	**GG**	GG	**CC**	**GG**
*004:01	*014	1	GG	GG	**CC**	CC	**AG**	**AG**	AA	AA	**CC**	**AG**	**CC**	**CC**	GG	**GG**	**GG**	GG	**CC**	**AG**
*005:02/*010	*014	1	GG	GG	**CC**	CC	**GG**	**AG**	AA	AA	**CC**	**AG**	**CC**	**CC**	GG	**GG**	**GG**	GG	**CC**	**AG**
*003	*004:01	1	GG	GG	**CC**	CC	**GA**	**AA**	AA	AA	**CC**	**GG**	**CC**	**CT**	GG	**GG**	**CC**	GG	**CC**	**GG**
*003	*009N	1	GG	GG	**CC**	CC	**GG**	**AA**	AA	AA	**CC**	**GG**	**CT**	**TT**	GG	**GG**	**CC**	GG	**CC**	**GG**
*005:03	*005:01	1	GG	GG	**CC**	CC	**GG**	**AA**	AA	AA	**CC**	**GG**	**CC**	**CC**	GG	**AG**	**GG**	GG	**CT**	**GG**
*004:01	*024	1	GG	GG	**CT**	CC	**AA**	**AA**	AA	AA	**CC**	**GG**	**CC**	**CC**	GG	**GG**	**GG**	GG	**CC**	**GG**

*Bold letters showed three genotypes or one heterozygote and one homozygote.

Genotyping by ASPE on microarrays was fully concordant with *MICB* genotyping using PCR-SBT in the sample-to-sample comparison (Table 6). MICB*002:01 and *004:01 alleles, which were determined using ASPE on microarrays, were identified as MICB*002:01:01 and MICB*004:01:01 alleles by PCR-SBT. Our SBT method could discriminate MICB*010 allele from MICB*005:02 allele because these two alleles are different in exon 6. We could identify only MICB*005:02 allele in Korean population using PCR-SBT (n = 99). However, we showed MICB*005:02/*010 allele because the primer sets used to amplify templates for ASPE on microarrays were not designed to differentiate MICB*005:02 allele from MICB*010 allele in exon 6. MICB*018 and *019 alleles, which have not previously been reported in Koreans, were found in this study.

**Table 6:** *MICB* alleles by ASPE on microarrays and PCR-SBT and *MICB* population allele frequency (Korean population) to compare them with the test sample frequency.

ASPE on microarrays	PCR-SBT (exons 2–6)	No.	ASPE on microarrays (2n = 400, %)	Cha et al [16], Korean population (2n = 278, %)
MICB*002:01	MICB*002:01:01	54	13.5	11.5
MICB*003	MICB*003	15	3.8	2.5
MICB*004:01	MICB*004:01:01	46	11.5	8.3
MICB*005:02/*010	MICB*005:02^¶^	222	55.5	57.2
MICB*005:03	MICB*005:03	21	5.3	8.3
MICB*008	MICB*008	21	5.3	6.8
MICB*009N	MICB*009N	11	2.8	2.2
MICB*014	MICB*014	8	2.0	3.2
MICB*018	MICB*018	1	0.3	
MICB*019	MICB*019	1	0.3	

^¶^Determination in *MICB* exon 6.

## Discussion

The ASPE method depends for identifying alleles on the sequence-specific extension of immobilized allele-specific extension primers that differ at the 3′ ends of their SNPs [11]. However, certain mismatches are not refractory to extension. The systemic characteristics and optimization of the ASPE procedure have been shown to be influenced by annealing temperature, template concentration, and Mg^2+^ concentration [18]. Of the extension primers examined in this study, which were designed to have a length of 18 to 21 bases, 14 showed excellent sensitivity and specificity in the control panel (Table 3).

Allele-specific PCR with *Taq* polymerase offers the greatest template discrimination (40- to 100-fold) against mismatches to thymine, guanine, or cytosine (T, G, or C) at the 3′ end of a primer, but not against mismatch to adenine (A) [19]. To improve the accuracy, we used the thermostable Thermo Sequenase which was engineered to catalyze the incorporation of dNTPs with higher efficiency than other DNA polymerases on SBE-based microarray [20]. For primers with cytosine (C) as the 3′ nucleotide, Thermo Sequenase was highly specific for template complementarity to this base (Table 4). By contrast, primers with thymine (T) at the 3′ end were less efficiently amplified regardless of the corresponding nucleotide on the template strand. These results are directly applicable to the design of primers for SNP detection and have made it possible to develop a general procedure to increase the specificity of extension primers by specifying an additional variable nucleotide at the 3′ end of the extension primer.

Different approaches have been proposed to improve the specificity of ASPE. As the specificity of primer-directed extension is not sufficient for quantitative SNP analysis, artificial mismatched bases have been introduced into the 3′ end regions of the specific primers as a way of improving the switching characteristics of the primer extension reactions. The best position in the primer for such artificial mismatched bases is the third position from the 3′ end of the primer [21]. In our control extension primers, mismatched control extension primers containing errors in the 2nd and 3rd bases from the 3′ end gave a completely negative signal, whereas primers containing an error in the 1st base from the 3′ end gave a weak signal (Fig 1). The specificity of extension primers targeting SNP region was also increased by the modification for refractory extension (Fig 2; Table 4). For further improvement of primer quality, apyrase, a nucleotide-degrading enzyme to the extension reaction, should be introduced to DNA microarrays [22, 23]. We did not use apyrase in our assay and the quality of primers was improved by optimal modifications instead. We retested the reactions if the MFI value was between 10,000 and 30,000. When the same values appeared over three trials, the results were confirmed using other methods such as PCR-SBT (data not shown).

Although we designed 36 primers to detect almost all of the known 41 *MICB* alleles, only 22 primers could be defined or improved using the control panel in this study. Therefore, we could not define the other rare alleles and it is necessary to confirm the quality of the 14 remaining primers using samples from other populations. In Korean population, the *MICB* alleles detected by the genotyping microarray were fully in concordance with those detected by PCR-SBT and we compared the *MICB* population allele frequency (Korean population) with the test sample frequency in Table 6 (2n = 400). Two of the alleles found in this study, MICB*018 and *019 alleles, had not been previously found in Koreans [16]. Therefore, it was possible to identify 13 *MICB* alleles in this study using verified allele-specific extension primers. Our microarray did not include SNP in exon 6 because these SNPs are present only in very rare alleles which have been not reported in population data of published papers and are not present even in 163 samples of the UCLA International Exchange Report program. Although we designed primers to detect SNPs in exon 2 to 5 of *MICB*, new alleles have recently been defined by SNPs in exon 6 [24]. MICB*010 and *005:02 alleles could not be discriminated by ASPE on microarrays because the primers had not been designed to differentiate MICB*005:02 allele from MICB*010 allele in exon 6.

The 13 *MICB* alleles identified by this method were MICB*002:01, *003, *004:01, *005:01, *005:02/*010, *005:03, *005:06, *008, *009N, *014, *018, *019 and *024 (Table 6). However, the rare 28 *MICB* alleles could not be identified; MICB*001, *005:04, *005:05, *005:07, *005:08, *006, *007, *011, *012, *015, *016, *017, *020, *021N, *022, *023, *025, *026, *027, *028, *029 and *030. Therefore, some *MICB* alleles can not discriminate and may result in error data.

If the frequency of the minor allele is greater than 1%, such variants are called polymorphisms. These 13 *MICB* alleles were found more than 1% in reported ethnic populations including Welsh, Spanish, and Chinese and the other alleles are present less than 1% only in particular populations [5, 10, 16, 24–26]. Therefore, this method may be useful for disease association studies in various populations, even though it can not detect rare alleles.

We had previously developed a typing method of the SNPs of cytokine genes using ASPE on a fluorescence bead array [27]. According to this study and the bead array study, we showed that allele typing could not only be possible on liquid phase but also on solid phase. Although Sanger and next generation sequencing is readily available in labs and new alleles can be detected, this DNA chip-based assay allows for a multiplex assay to detect several mutations in addition to a being relatively rapid and cost-effective means of detecting defined mutations for genetic diagnosis.

## Conclusions

We have established a system for genotyping *MICB* alleles using ASPE on microarrays. Available operation of control and allele-specific extension primers was representatively confirmed by schematic patterns of successful allele-specific extension primers that could discriminate 13 *MICB* alleles (S1 and S2 Figs). In conclusion, our method for genotyping *MICB* alleles using ASPE on microarrays could be applicable for large-scale SNP typing studies of population and disease associations.

## Supporting Information

S1 Fig Schematic patterns of 22 allele-specific primers that could discriminate 13 *MICB* alleles. (DOCX)

S2 Fig Available operation of successful allele-specific primers representatively confirmed by genotyping of 10 *MICB* alleles, using ASPE on microarrays. (DOCX)
