# Supplementary figures and images for: MICB Allele Genotyping on Microarrays by Improving the Specificity of Extension Primers
Source: PLoS One. 2015 Nov 16;10(11):e0142467. doi: 10.1371/journal.pone.0142467 (PMC4646348; doi:10.1371/journal.pone.0142467)

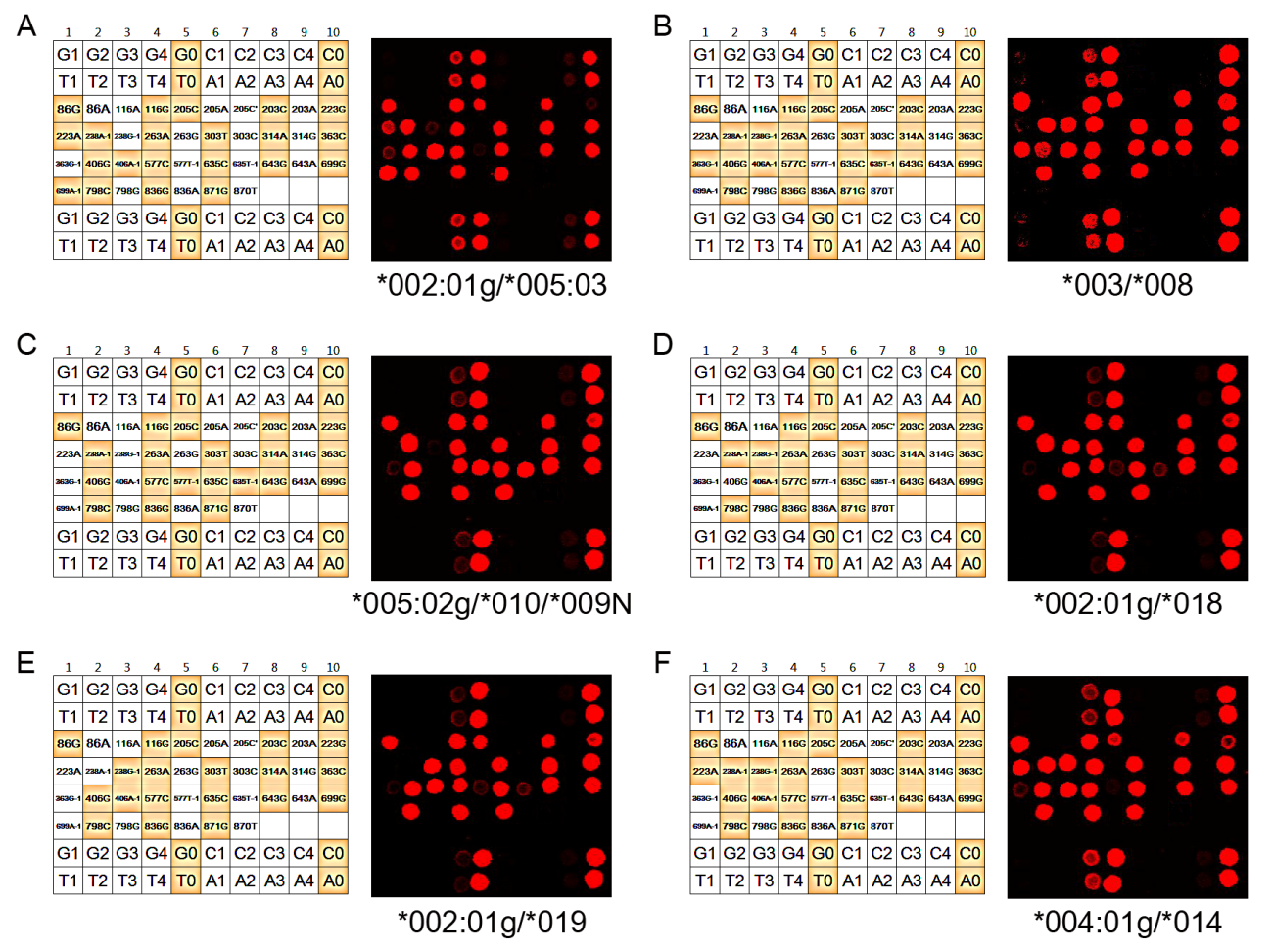

Supplement: S2 Fig — (DOCX) [file pone.0142467.s002.docx]
