# Supplementary material for: MICB Allele Genotyping on Microarrays by Improving the Specificity of Extension Primers
Source: PLoS One. 2015 Nov 16;10(11):e0142467. doi: 10.1371/journal.pone.0142467 (PMC4646348; doi:10.1371/journal.pone.0142467)
Supplement: Supplementary file 2 [file pone.0142467.s001.docx]

Allele-specific primers	*MICB**005:02g/*010	*MICB**003	*MICB**009N	*MICB**005:06	*MICB**005:01	*MICB**004:01	*MICB**024	*MICB**005:03	*MICB**014	*MICB**008	*MICB**002:01	*MICB**018	*MICB**019	Allele-specific primers
MICB 406G	G	G	G	G	G	G	G	G	A	A	A	A	G	*MICB* 406A-2
MICB 238A-1	A	A	A	A	A	A	A	A	G	G	G	A	G	*MICB* 238G-1
MICB 635C	C	T	T	C	C	C	C	C	C	C	C	C	C	*MICB* 635T-2
MICB 577C	C	C	T	C	C	C	C	C	C	C	C	C	C	*MICB* 577T-1
MICB 762G	G	G	G	T	G	G	G	G	G	G	G	G	G	*MICB* 762T
MICB 870C	C	C	C	C	T	C	C	C	C	C	C	C	C	*MICB* 870T
MICB 223G	G	G	G	G	G	A	G	G	G	G	G	G	G	*MICB* 223A
MICB 163C	C	C	C	C	C	C	T	C	C	C	C	C	C	*MICB* 163T
MICB 699G	G	G	G	G	G	G	G	A	G	G	G	G	G	*MICB* 699A-1
MICB 871G	G	G	G	G	G	G	G	G	A	G	G	G	G	*MICB* 871A
MICB 363C	C	C	C	C	C	C	C	C	C	G	C	C	C	*MICB* 363G-1
